# Supplementary material for: Sedimentary pyrite sulfur isotopes track the local dynamics of the Peruvian oxygen minimum zone
Source: Nat Commun. 2021 Jul 20;12:4403. doi: 10.1038/s41467-021-24753-x (PMC8292381; doi:10.1038/s41467-021-24753-x)
Supplement: Supplementary file 1 — Supplementary information [file 41467_2021_24753_MOESM1_ESM.pdf]

Sedimentary pyrite sulfur isotopes track the local dynamics of the Peruvian Oxygen Minimum Zone

Supplementary information

This PDF file includes:

Supplementary Note
Figs. S1 to S8
Tables S1
References #01-23

Supplementary Note. 1

Sedimentary setting

Samples from ODP Leg 201 – site 1229E were analyzed in this study (Supplementary Fig. 1). The drilling site is in the Salaverry basin on the shallow continental shelf of the Peru Margin, located within the modern OMZ at ~150 m water depth, where oxygen concentrations in bottom waters are between >5 µmol L^{-1} (refs 1, 2, 3; Supplementary Fig. 7). Strong offshore Ekman transport leads to coastal upwelling of cold, nutrient-rich water into the upper thermocline, promoting high surface primary productivity along the western edge of the Peru margin[4, 5, 6]. The resulting enhanced flux of sinking organic matter leads to rapid depletion of oxygen in the water column and elevated delivery of organics to the sediments of the continental shelf. As a result, the region is characterized by an intense OMZ that impinges on the continental shelf between ~50 and 500 m water depth[7].

All samples investigated in the present study are from the core E from the depth interval between 0 and 44 m. Sediments are characterized by alternations of olive-green well-laminated diatom ooze and a silty-clay diatom ooze with elevated organic matter content. According to Skilbeck and Fink (2001), two main erosional surfaces associated with coarser silt and fine sand are present in Hole A, 201-1229A-2H-6 and 201-1229A-6H-2. After careful cross-correlation of the magnetic susceptibility in hole A, D and E (Supplementary Fig. 1) it turns out that the section 201-1229A-6H-2 is located below 45 m and is, therefore, not within the depth interval studied here. Close visual (picture) examination of the core 201-1229A-2H-6 reveals the presence of a grey clay

layer, but unlike in the deeper sections reported in Skilbeck and Fink (2001; e.g., 201-1229A-11H-6 or 201-1229A-14H-4), there is no evidence of shell debris or phosphatic hard ground, which are characteristic erosional features at the study site. Furthermore, according to our age model and the one developed by Schrader (1992) for Hole 681A (same drilling site), irrespective of whether one performs a depth cross-correlation, this section seems to be deposited during an interglacial interval. Hence, it is unlikely that an erosional feature affected the hole location during a period of high sea level. Instead, we propose that the greenish facies indicate a period of forced regression, without erosion at the study site, during the MIS7 lowstand.

Porewater chemistry reveals sulfate depletion through the top 30 m below sea-floor (mbsf) with a upper sulfate-methane transition zone (SMTZ) between 25 and 35 mbsf and a deep SMTZ around 90 mbsf[8]. Dissolved sulfide was detected throughout the entire core attesting to ongoing microbial sulfate reduction[9].

FIGURES

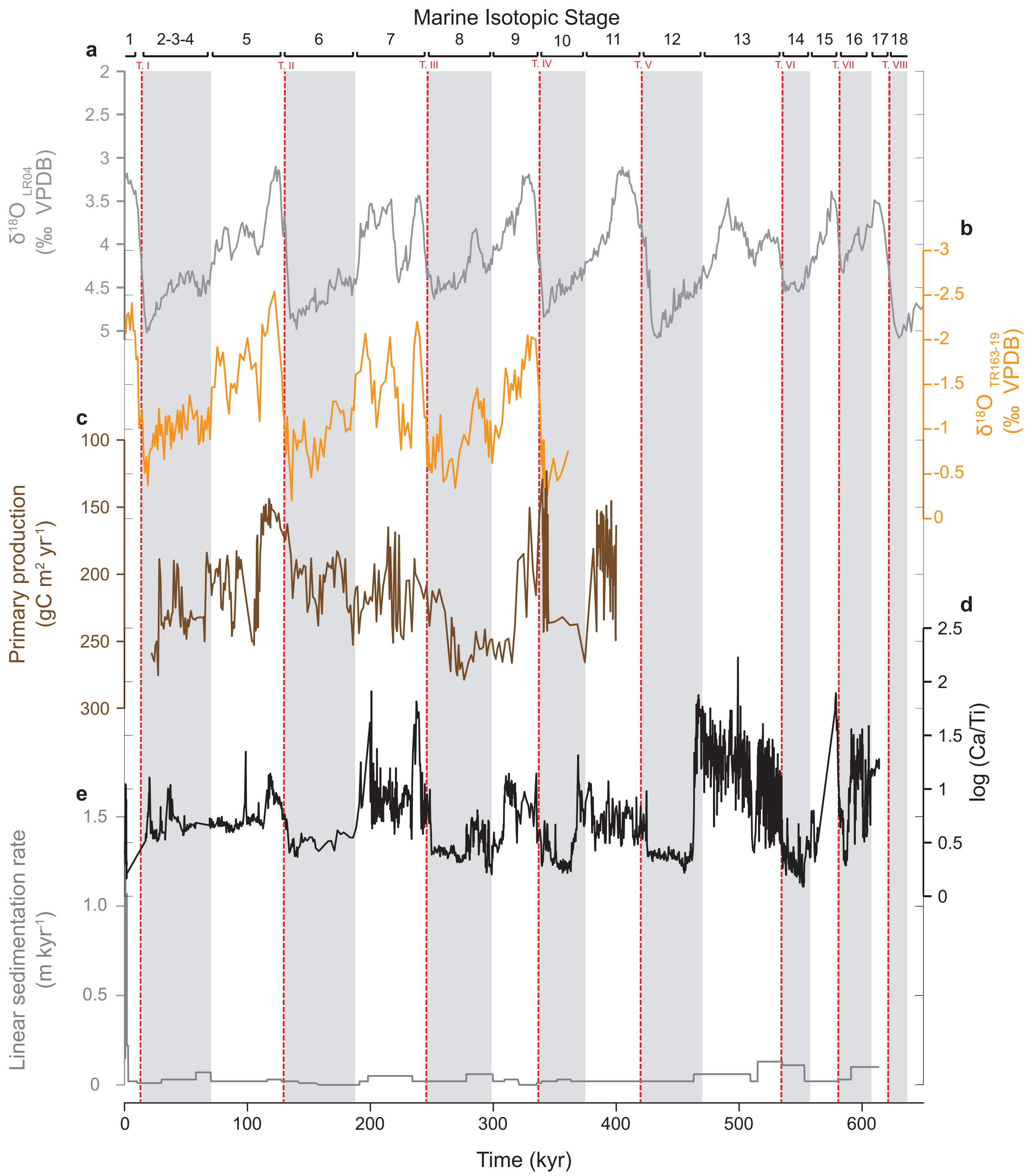

Supplementary Fig. 1: Age model construction. Shown are (**a**) Reference benthic oxygen isotope record LR04 from ref. 10; (**b**) the reference $\delta^{18}O$ of planktonic foraminifera (*G. ruber*) from the Eastern Pacific TR163-19 core, published in ref. 11; (**c**) the reference primary production reconstruction (based on marine diatom assemblage) from the ODP 112 site 681A, published in

ref. 12; (**d**) log(Ca/Ti) used to track glacial and interglacial periods (data from ref. 13); (**e**) linear sedimentation rate as reconstructed from our depth-age correlation. Grey vertical bands represent glacial intervals, white bands correspond to interglacial periods. Vertical red dashed lines reflect glacial terminations (T) with timing according to ref. [14]. Grey dashed vertical lines reflect marine stages according to ref. [15].

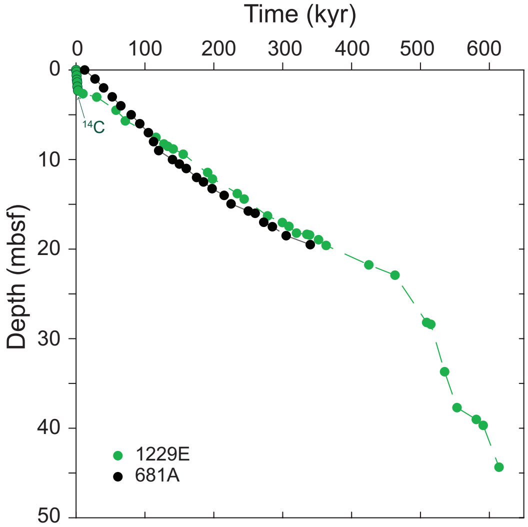

Supplementary Fig. 2: Age model comparison between this study (green dots and dashed line) and previously developed age-depth correlation in the ODP 112 site 681A (same location, black dots) from ref. 12.

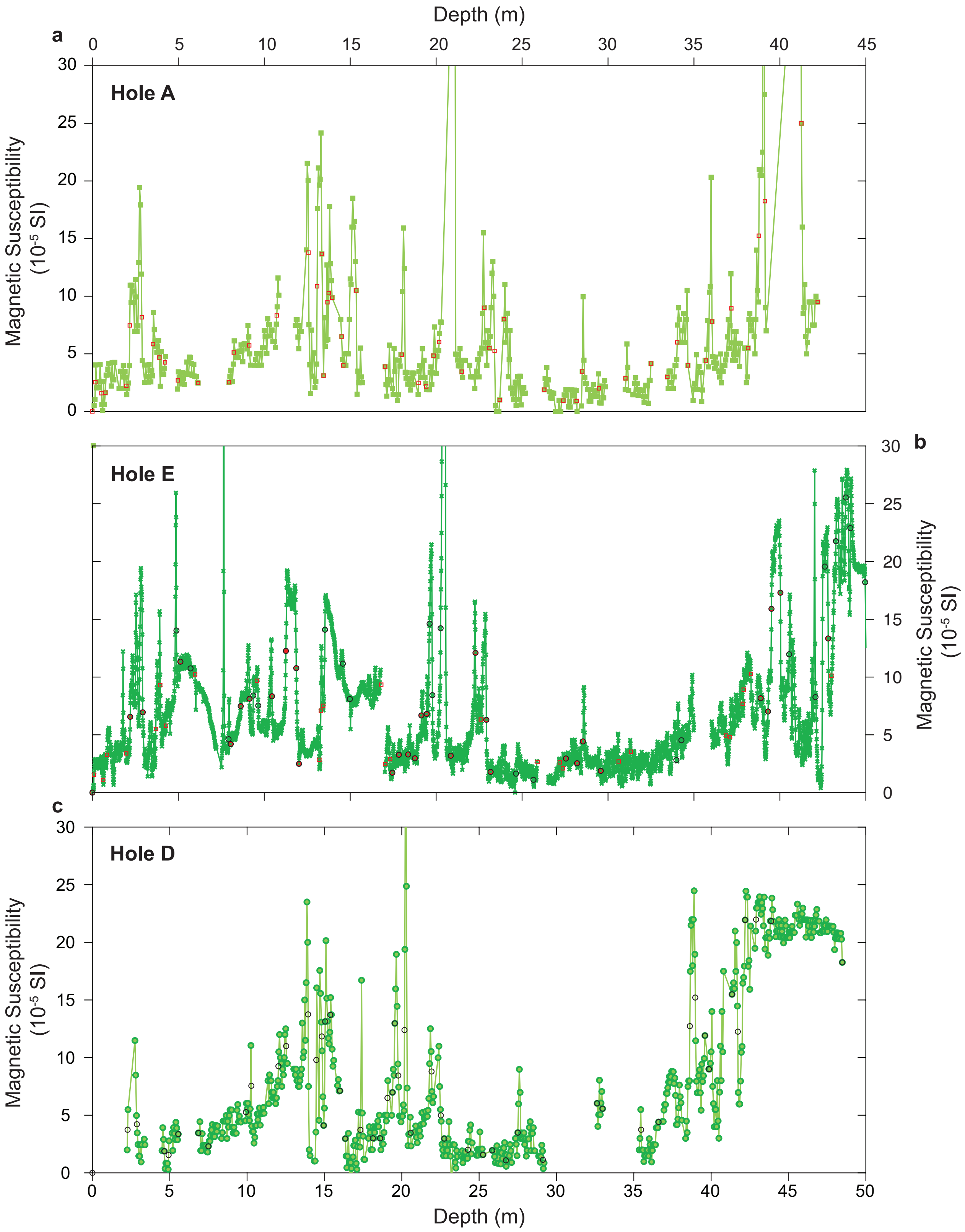

Supplementary Fig. 3: Site-to-site correction. Shown is the magnetic susceptibility in (**a**) ODP 201-1229A; (**b**) ODP 201-1229E and (**c**) ODP 201-1229D. Red squares and black circles show the

depth correlation between holes A-E and holes D-E, respectively.

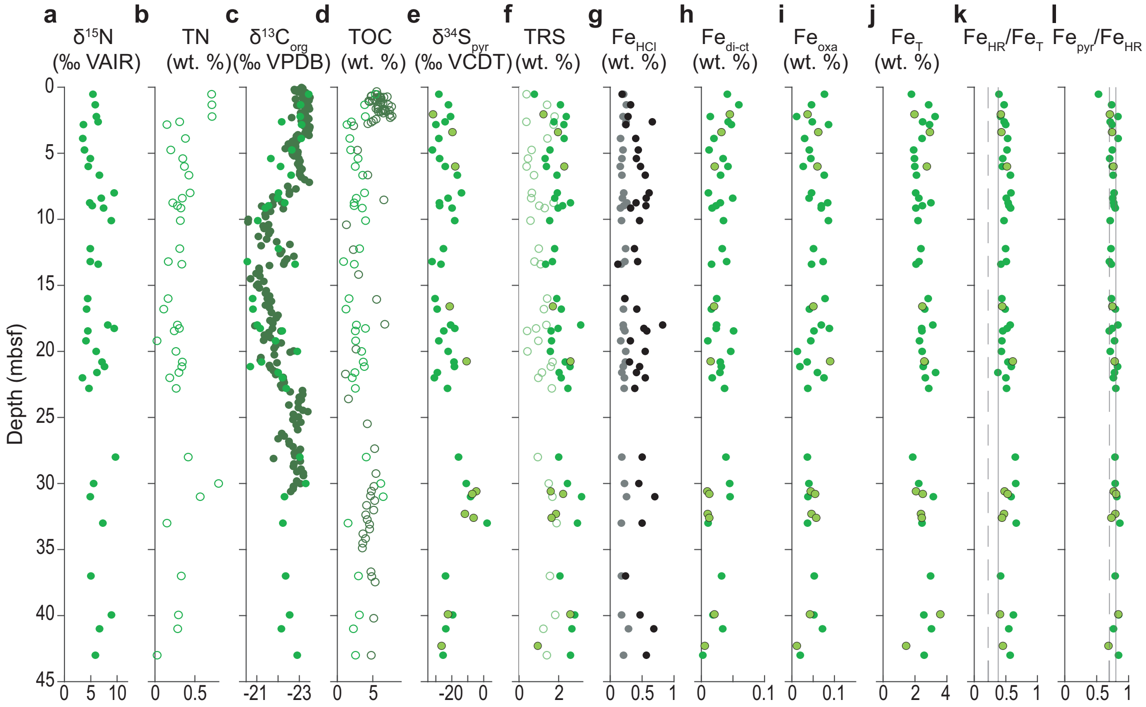

Supplementary Fig. 4: ODP Leg 201 – site 1229 depth profiles of geochemical records. (**a**) $\delta^{15}N$ from this study (green dots); (**b**) Total Nitrogen (TN) from this study (green circles); (**c**) $\delta^{13}C_{org}$ from this study (green dots) and from ref. 8 (dark green dots); (**d**) Total organic carbon (TOC) from this study (green circles) and from ref. 8 (dark green circles); (**e**) $\delta^{34}S_{pyr}$ from this study (green dots) and from ref. [16] (pale green dots); (**f**) Total reduced sulfur (TRS) from this study (green circles) and prior to post-sampling oxidation (green dots) determined by adding Fe^{III}_{HCl} and Fe_{CRS} - also shown are TRS from ref. [16] (pale green dots); (**g**) HCl-extracted Fe^{II} (light black dots) represents unsulfidized Fe^{II} and HCl-extracted Fe^{III} (black dots) represents metastable Fe^{III} hydroxides, interpreted here to reflect post-coring oxidation of AVS and pyrite; (**h**) Dithionite-extracted Fe^{III} (Fe_{di-ct}) represents crystalline Fe^{III} (oxyhydr)oxides (green dots) and from ref. [16] (pale green dots); (**i**) Oxalate-extracted Fe (Fe_{oxa}) represents magnetite (green dots) and from ref. [16] (pale green dots); (**j**) Total Fe (green dots) and from ref. [16] (pale green dots); (**k**) Highly reactive ($Fe_{HR} = Fe^{III}_{HCl} + Fe_{di-ct} + (Fe_{CRS} + Fe^{II}_{HCl}) + Fe_{oxa}$) over Total Fe (green dots) and from ref. [16] (pale green dots); (**l**) The iron sulfide pool ($Fe_{CRS} + Fe^{II}_{HCl}$) prior to post-sampling oxidation over Highly reactive Fe (green dots) and from ref. [16] (pale green dots).

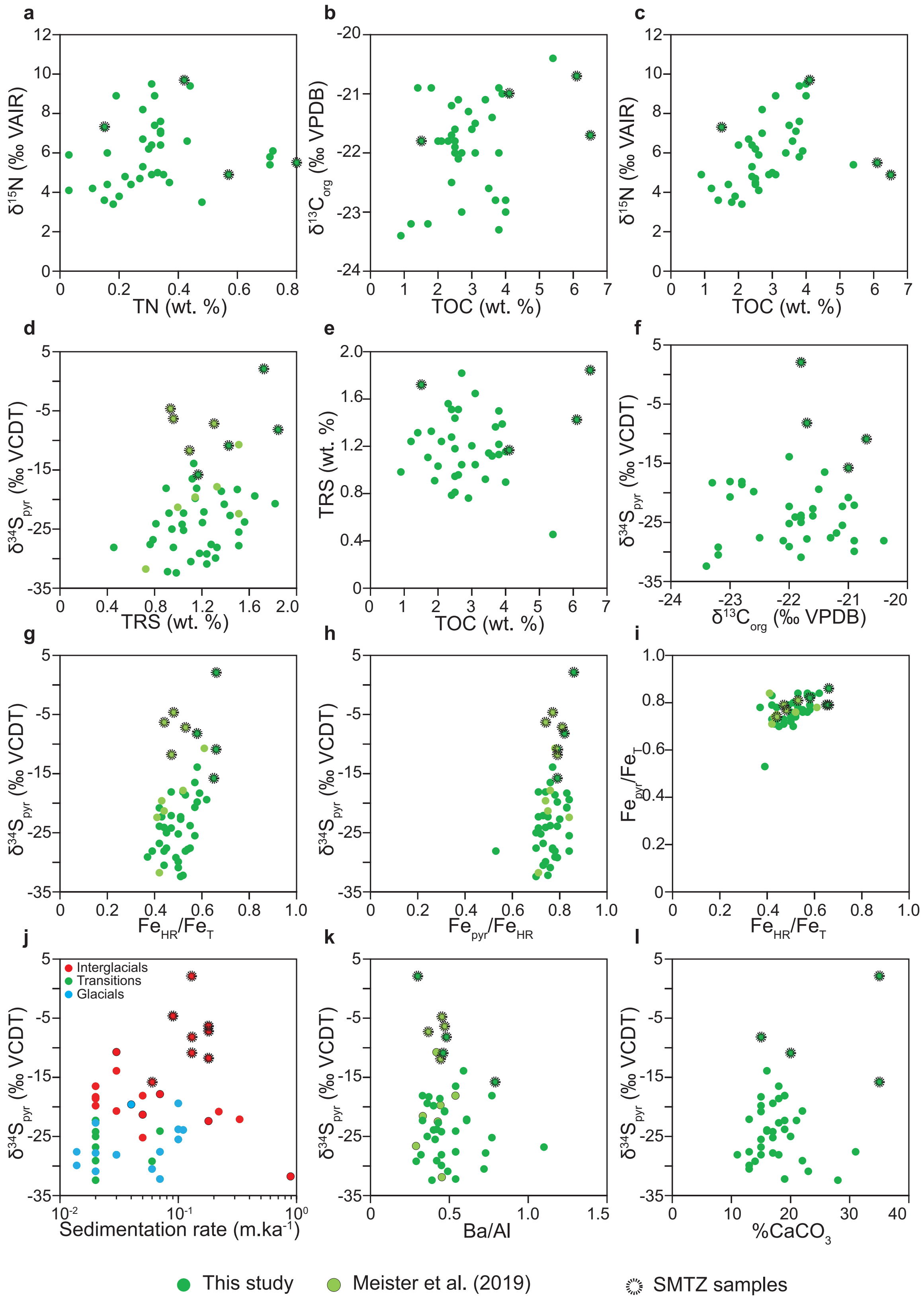

Supplementary Fig. 5: Geochemical and isotopic data from ODP leg 201 – hole 1229. (**a**) $\delta^{15}N$

vs. total nitrogen (TN); (**b**) $\delta^{13}C_{org}$ vs. total organic carbon (TOC); (**c**) $\delta^{15}N$ vs. total organic carbon (TOC); (**d**) $\delta^{34}S$ vs. total reduced sulfur (TRS); (**e**) Total reduced sulfur (TRS) vs. total organic carbon (TOC); (**f**) $\delta^{34}S$ vs. $\delta^{13}C_{org}$, (**g**) $\delta^{34}S$ vs. Fe_{HR}/Fe_T ratio; (**h**) $\delta^{34}S$ vs. Fe_{PYR}/Fe_{HR} ratio; (**i**) Fe_{HR}/Fe_T vs. Fe_{HR}/Fe_T ratio; (**j**) $\delta^{34}S$ vs. sedimentation rate in m ka^{-1}; (**k**) $\delta^{34}S$ vs. Ba/Al (an indicator of the sulfate methane transition zone, SMTZ); and (**l**) $\delta^{34}S$ vs. $CaCO_3$.

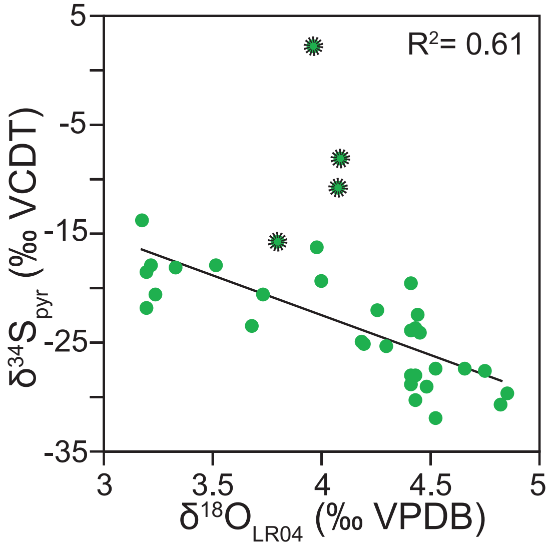

Supplementary Fig. 6: $\delta^{18}O_{LR04}$- $\delta^{34}S_{pyr}$ cross-plot. The three samples located within the modern sulfate-methane transition zone (SMTZ; dotted black outline) are excluded from the linear regressions of $\delta^{34}S_{pyr}$ on $\delta^{18}O_{LR04}$. Oxygen isotope data are from ref. 10.

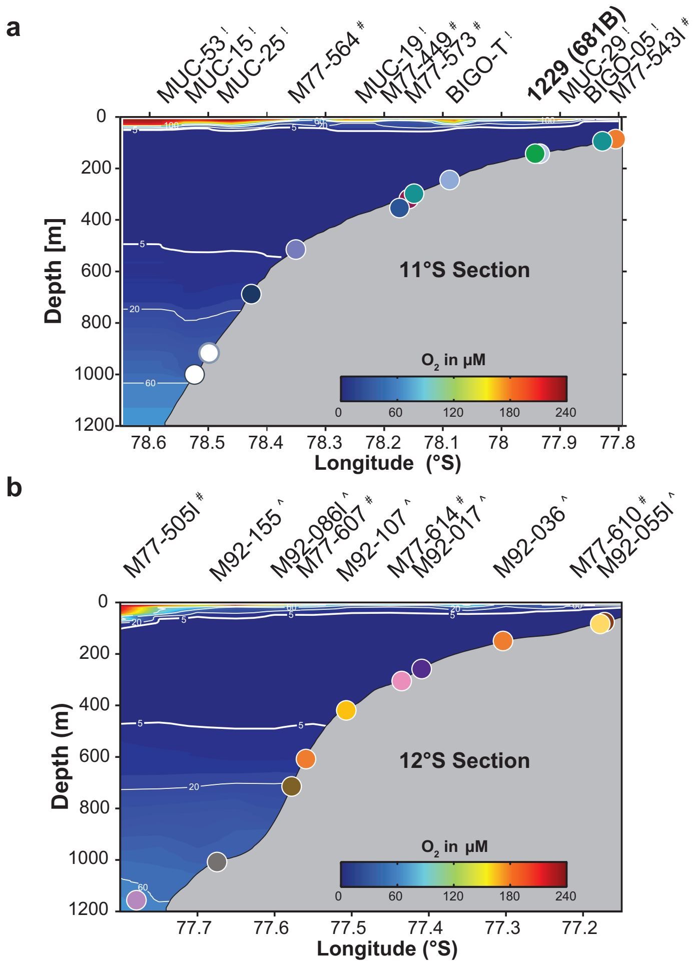

Supplementary Fig. 7: Slope bathymetry profiles and cross-sections of dissolved oxygen concentration (µM) published in ref. 17 at 11°S (top) and 12°S (bottom). Also shown are the location of cores previously published over the Peru margin. Symbols denote references in which the core chemistry (solid or porewater) were published: # to ref. 18, ^ to ref. 19, ! to ref. 20.

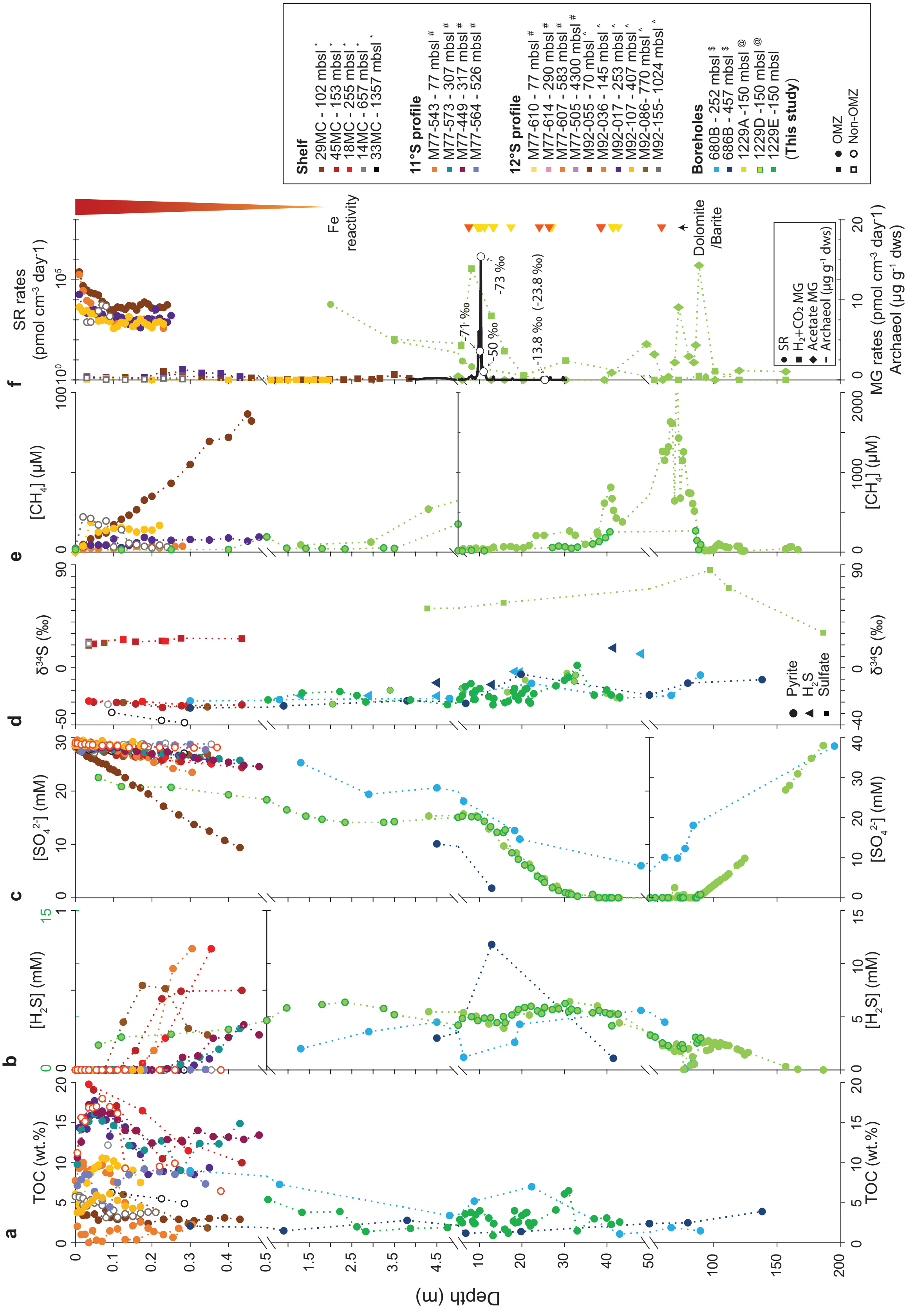

a
b
c
d
e
f
Depth (m)
TOC (wt.%)
[H2S] (mM)
[SO42-] (mM)
δ34S (‰)
[CH4] (µM)
SR rates (pmol cm-3 day-1)
MG rates (pmol cm-3 day-1)
Archaeol (µg g-1 dws)
Pyrite
H2S
Sulfate
SR
H2+CO2 MG
Acetate MG
Archaeol (µg g-1 dws)
Fe reactivity
Dolomite /Barite
-71 ‰
-50 ‰
-73 ‰
-13.8 ‰ (-23.8 ‰)
Shelf
29MC - 102 mbsl *
45MC - 153 mbsl *
18MC - 255 mbsl *
14MC - 657 mbsl *
33MC - 1357 mbsl *
11°S profile
M77-543 - 77 mbsl #
M77-573 - 307 mbsl #
M77-449 - 317 mbsl #
M77-564 - 526 mbsl #
12°S profile
M77-610 - 77 mbsl #
M77-614 - 290 mbsl #
M77-607 - 583 mbsl #
M77-505 - 4300 mbsl #
M92-055 - 70 mbsl ^
M92-036 - 145 mbsl ^
M92-017 - 253 mbsl ^
M92-107 - 407 mbsl ^
M92-086 - 770 mbsl ^
M92-155 - 1024 mbsl ^
Boreholes
680B - 252 mbsl $
686B - 457 mbsl $
1229A -150 mbsl @
1229D -150 mbsl @
1229E -150 mbsl
(This study)
OMZ
Non-OMZ

Supplementary Fig. 6: Peruvian shelf geochemical records compilation. Shown are (**a**) Total organic carbon (TOC); (**b**) Aqueous sulfide concentration; (**c**) Aqueous sulfate in porewater concentration; (**d**) $\delta^{34}S$ of aqueous sulfate (triangles) and pyrite (dots); (**e**) Methane concentration in porewater; (**f**) Rate of sulfate reduction (refs. 9, 19; top axis) and methanogenesis (refs. 9, 19; bottom axis) with archaeol concentration (ref. 13; black line) and $\delta^{13}C_{archeaol}$ (refs. 13, 21; dots). The colors and symbols are as follows: @ refers to ref. 16, # to ref. 18, ^ to ref. 19, * to ref. 22, $ to ref. 23. Locations of the cores are in Fig. 1a. Please note discontinuous depth scale in panels b, c and e.

TABLE

Supplementary Table 1: Depth, estimated age, and associated geochemical information from samples from the ODP 201 – site 1229 borehole E.

TABLE DR1. GEOCHEMICAL RESULTS OF ODP 201 - SITE 1229 - HOLE E

Depth	Age	CaCO3	Ba/Al	δ15N	TN	δ13Corg	TOC	δ34Spyr	TS	Fe^{II}_{HCl}	Fe^{III}_{HCl}	Fe_{di-ct}	Fe_{oxa}	Fe_{CRS}	$Corr.Fe_{pyr}$	Fe_T
(mbsf)	(ka)	(wt. %)		(‰ VAIR)	(wt. %)	(‰ VPDB)	(wt. %)	(‰ VCDT)	(wt. %)	(wt. %)	(wt. %)	(wt. %)	(wt. %)	(wt. %)	(wt. %)	(wt. %)
0.53	0.76	15		5.4	0.7	-20.4	5.4	-28.1	1.4	0.21	0.19	0.04	0.08	0.18	0.37	1.78
1.33	1.6	13	0.61	5.8	0.7	-20.9	3.8	-22.1	1.4	0.25	0.32	0.06	0.05	0.67	0.99	2.86
2.22	2.7	15	0.47	6.1	0.7	-21.0	3.9	-20.8	1.8	0.20	0.28	0.01	0.01	0.85	1.13	3.26
2.62	9.2	17	0.45	6.4	0.3	-21.8	2.0	-24.2	0.4	0.25	0.66	0.04	0.05	0.18	0.84	2.48
2.83	13.3	13	0.45	3.6	0.1	-20.9	1.4	-29.9	1.8	0.24	0.25	0.05	0.09	0.83	1.07	2.91
3.88	34.0	11	0.45	3.5	0.5	-20.9	1.8	-28.1	1.5	0.16	0.40	0.02	0.03	0.68	1.08	2.44
4.75	59.9	19	0.54	3.8	0.2	-25.4	1.9	-32.2	0.6	0.20	0.44	0.01	0.04	0.30	0.74	1.92
5.40	64.7	13	0.54	4.9	0.3	-21.3	2.9	-27.6	0.5	0.18	0.41	0.03	0.04	0.21	0.62	1.97
6.00	69.1	16	0.54	4.5	0.4	-21.9	2.5	-24.1	0.4	0.16	0.47	0.04	0.03	0.18	0.66	1.99
6.66	87.7	18	0.54	6.6	0.4	-21.4	3.6	-16.5	0.8	0.18	0.55	0.03	0.08	0.36	0.91	2.09
8.00	123.5	16	0.59	9.4	0.4	-22.0	3.8	-13.9	0.7	0.21	0.61	0.01	0.05	0.31	0.92	2.04
8.40	130.5	21	0.61	7.0	0.3	-22.0	2.7	-22.3	0.6	0.19	0.56	0.05	0.04	0.29	0.85	2.24
8.75	139.1	17	0.73	4.8	0.2	-21.7	2.4	-27.8	1.8	0.26	0.41	0.03	0.08	0.83	1.23	3.00
8.97	155.5	31	0.54	5.3	0.3	-22.5	2.4	-27.6	1.0	0.21	0.56	0.02	0.07	0.48	1.04	2.46
9.15	192.0	15	0.40	7.4	0.3	-22.6	3.5	-19.8	1.3	0.16	0.32	0.02	0.07	0.61	0.93	2.05
10.10	204.9	19	0.77	8.9	0.3	-23.0	4.0	-18.1	0.6	0.18	0.46	0.04	0.09	0.27	0.73	2.19
12.22	225.7	17	0.77	4.9	0.3	-22.0	3.1	-25.2	1.0	0.24	0.38	0.03	0.05	0.47	0.85	2.37
13.20	238.1	28	0.39	4.9	0.6	-23.4	0.9	-32.4	0.8	0.23	0.43	0.04	0.07	0.37	0.80	2.25
13.40	244.3	15	1.10	6.4	0.3	-21.2	2.4	-26.8	1.1	0.17	0.12	0.02	0.05	0.52	0.64	2.07
16.00	290.6	13	0.72	4.4	0.2	-23.2	1.7	-30.5	1.4	0.23	0.23	0.02	0.08	0.67	0.90	2.83
16.80	297.5	14	0.29	4.2	0.1	-23.2	1.2	-29.2	1.3	0.21	0.42	0.02	0.04	0.60	1.01	2.61
18.00	316.9	22	0.47	8.2	0.3	-23.0	2.7	-20.7	1.4	0.20	0.82	0.02	0.07	0.66	1.48	3.12
18.26	325.4	19	0.33	9.5	0.3	-22.8	4.0	-18.1	0.9	0.21	0.53	0.02	0.09	0.41	0.94	2.42
18.45	339.7	20	0.36	4.4	0.2	-21.8	2.5	-25.0	0.4	0.23	0.57	0.05	0.05	0.19	0.77	2.44
19.20	356.3	18	0.32	4.1	0.0	-22.1	2.6	-28.1	1.0	0.17	0.32	0.01	0.04	0.46	0.78	2.28
20.00	374.7	19	0.33	6.0	0.2	-21.1	3.4	-22.3	0.4	0.24	0.55	0.05	0.01	0.20	0.75	2.45
20.80	397.6	18	0.44	7.1	0.3	-22.8	3.7	-18.6	1.7	0.24	0.30	0.03	0.04	0.80	1.11	2.64
21.15	407.6	15	0.37	7.6	0.3	-23.3	3.8	-18.3	1.6	0.20	0.46	0.03	0.02	0.76	1.22	2.52
21.60	420.4	22	0.42	6.2	0.3	-22.0	2.5	-29.1	1.2	0.18	0.41	0.03	0.06	0.55	0.96	3.29
22.00	432.9	23	0.49	3.4	0.2	-21.8	2.1	-30.9	1.0	0.22	0.55	0.02	0.08	0.46	1.01	2.65
22.80	459.4	18	0.43	4.7	0.3	-21.6	2.5	-22.7	1.7	0.22	0.39	0.04	0.04	0.79	1.17	2.86
28.00	507.3	35	0.79	9.7	0.4	-21.0	4.1	-15.8	1.0	0.18	0.50	0.04	0.04	0.45	0.95	1.86
30.00	521.0	20	0.46	5.5	0.8	-20.7	6.1	-10.9	1.5	0.22	0.45	0.04	0.04	0.71	1.16	2.24
31.00	524.8	15	0.48	4.9	0.6	-21.7	6.5	-8.2	1.7	0.26	0.70	0.05	0.04	0.80	1.50	3.15
33.00	532.4	35	0.30	7.3	0.2	-21.8	1.5	2.1	1.9	0.17	0.50	0.01	0.04	0.90	1.40	2.44
37.00	549.9	16	0.40	5.0	0.3	-21.6	3.0	-23.9	1.6	0.18	0.24	0.03	0.05	0.74	0.98	2.98
39.95	592.3	17	0.36	8.9	0.2	-21.5	3.1	-19.4	1.9	0.18	0.47	0.02	0.05	0.87	1.34	2.55
41.00	597.5	19	0.42	6.7	0.3	-21.8	2.3	-23.8	1.3	0.29	0.68	0.03	0.07	0.58	1.27	3.03
43.00	607.4	15	0.41	5.9	0.0	-21.1	2.6	-25.5	1.4	0.21	0.57	0.00	0.02	0.67	1.23	2.57

Supplementary References

1. Hamersley R*, et al.* Anaerobic ammonium oxidation in the Peruvian oxygen minimum zone. *Limnology and Oceanography* **52**, 923-933 (2007).

2. Scholz F, Siebert C, Dale AW, Frank M. Intense molybdenum accumulation in sediments underneath a nitrogenous water column and implications for the reconstruction of paleo-redox conditions based on molybdenum isotopes. *Geochimica et Cosmochimica Acta* **213**, 400-417 (2017).

3. Sommer S*, et al.* Depletion of oxygen, nitrate and nitrite in the Peruvian oxygen minimum zone cause an imbalance of benthic nitrogen fluxes. *Deep Sea Research Part I: Oceanographic Research Papers* **112**, 113-122 (2016).

4. Molina-Cruz A. The relation of the Southern Trade Winds to Upwelling Processes during the Last 75000 Years. *Quaternary Research* **8**, 324-338 (1977).

5. Brockmann C, Fahrbach E, Huyer A, Smith RL. Poleward undercurrent along the Peru coast 5 to 15°S. *Deep Sea Research* **27A**, 847-856 (1980).

6. Gutiérrez D*, et al.* Oxygenation episodes on the continental shelf of central Peru: Remote forcing and benthic ecosystem response. *Progress in Oceanography* **79**, 177-189 (2008).

7. Suess E, Huene Rv. Ocean Drilling Pragram Leg 112, Peru continental margin: Part 2, Sedimentary history and diagenesis in a castal upwelling environment. *Geology* **16**, 939-943 (1988).

8. D'Hondt SL, Jørgensen BB, Miller DJ. Shipboard scientific party _ 10. Site 1229. *Proceedings of the ocean Drilling Programs, Initial Reports* **201**, (2003).

9. D'Hondt S, Rutherford S, Spivack AJ. Metabolic activity of subsurface life in deep-sea sediments. *Science* **295**, 2067-2070 (2002).

10. Lisiecki LE, Raymo ME. A Pliocene-Pleistocene stack of 57 globally distributed benthic δ18O records. *Paleoceanography* **20**, 1-17 (2005).

11. Lea DW, Pak DK, Spero HJ. Climate impact of late quaternary equatorial pacific sea surface temperature variations. *Science* **289**, 1719-1724 (2000).

12. Schrader H. Coastal upwelling and atmospheric CO2 changes over the last 400,000 years: Peru. *Marine Geology* **107**, 239-248 (1992).

13. Contreras S*, et al.* Cyclic 100-ka (glacial-interglacial) migration of subseafloor redox zonation on the Peruvian shelf. *Proc Natl Acad Sci U S A* **110**, 18098-18103 (2013).

14. Barker S*, et al.* 800,000 years of abrupt climate variability. *Science* **334**, 347-351

(2011).

15. Railsback LB, Gibbard PL, Head MJ, Voarintsoa NRG, Toucanne S. An optimized scheme of lettered marine isotope substages for the last 1.0 million years, and the climatostratigraphic nature of isotope stages and substages. *Quaternary Science Reviews* **111**, 94-106 (2015).

16. Meister P, Brunner B, Picard A, Bottcher ME, Jorgensen BB. Sulphur and carbon isotopes as tracers of past sub-seafloor microbial activity. *Sci Rep* **9**, 604 (2019).

17. Dale AW*, et al.* Organic carbon production, mineralisation and preservation on the Peruvian margin. *Biogeosciences* **12**, 1537-1559 (2015).

18. Pfannkuche O. Climate- Biogeochemistry interactions in the tropical ocean of the SE-American oxygen minimum zone. In: *METEOR Cruise No. 77 Leg 1*). Leibniz-Institut für Meereswissenschaften, IFM-GEOMAR Kiel, Germany (2008).

19. Maltby J, Sommer S, Dale AW, Treude T. Microbial methanogenesis in the sulfate-reducing zone of surface sediments traversing the Peruvian margin. *Biogeosciences* **13**, 283-299 (2016).

20. Scholz F, Severmann S, McManus J, Noffke A, Lomnitz U, Hensen C. On the isotope composition of reactive iron in marine sediments: Redox shuttle versus early diagenesis. *Chemical Geology* **389**, 48-59 (2014).

21. Biddle JF*, et al.* Heterotrophic Archaea dominate sedimentary subsurface ecosystems off Peru. *Proc Natl Acad Sci U S A* **103**, 3846-3851 (2006).

22. Böning P*, et al.* Geochemistry of Peruvian near-surface sediments. *Geochimica et Cosmochimica Acta* **68**, 4429-4451 (2004).

23. Mossmann J, Aplin AC, Curtis C, Coleman ML. Geochemistry of inorganic and organic sulphur in organic-rich sediment from the Peru margin. *Geochimica et Cosmochimica Acta* **55**, 3581-3595 (1991).
